# Albumin-to-creatinine ratio as a predictor of all-cause mortality and hospitalization of congestive heart failure in Chinese elder hypertensive patients with high cardiovascular risks

**DOI:** 10.1186/s40885-018-0095-3

**Published:** 2018-08-15

**Authors:** Mingming Liu, Yan Liang, Jun Zhu, Yanmin Yang, Wenfang Ma, Guozheng Zhang

**Affiliations:** 1 State Key Laboratory of Cardiovascular Disease, Emergency and Critical Care Center, National Center for Cardiovascular Diseases, Fuwai Hospital, Chinese Academy of Medical Sciences and Peking Union Medical College, No. 167, Beilishi Road, Xicheng District, Beijing, 100037 China; 2 Department of Emergency Medicine, Tongji Hospital, Tongji Medical College, Huazhong University of Science and Technology, Jiefang Avenue No. 1095, Wuhan, 430030 China

**Keywords:** Albumin-to-creatinine ratio, Cardiovascular disease, Aging, Mortality, Heart failure hospitalization

## Abstract

**Background:**

Data are limited with regard to the relationship of albuminuria and major adverse cardiovascular events (MACE) in Chinese elder patients with high cardiovascular risk.

**Methods:**

We did a retrospective cohort study using Chinese elder patients with high cardiovascular risks (*n* = 1474) to identify the association of albumin-to-creatinine ratio (ACR) and the incidence of MACE and all-cause mortality. Individuals were followed up from January, 2002 to November, 2007. The all-cause mortality and MACE, composite outcome of cardiovascular death, myocardial infarction, stroke and hospitalization of congestive heart failure were defined as primary endpoint.

**Results:**

During the median following up of 56 months, 213 patients developed primary endpoint and 117 patients died. Patients with higher baseline urinary ACR (> 30 mg/g) experienced a nearly 2-fold of all-cause mortality and a 3-fold of heart failure hospitalization than those with lower baseline urinary ACR (≤10 mg/g).MACE, cardiovascular death, stoke and myocardial infarction showed no difference in three grades of urinary ACR (> 30 mg/g, 10 mg/g-30 mg/g, ≤10 mg/g) in this cohort. Patients above 65 years with increased ACR tended to experience higher mortality risks, and the association of increased ACR with higher hospitalization of congestive heart failure seemed to be more prominent in patients below 65 years than above 65 years.

**Conclusions:**

In this post hoc analysis of Chinese individuals with high cardiovascular risks, higher urinary ACR was associated with higher all-cause mortality and heart failure hospitalization. Further studies are needed to find out whether there is age-specific ACR cutoff point.

**Electronic supplementary material:**

The online version of this article (10.1186/s40885-018-0095-3) contains supplementary material, which is available to authorized users.

## Background

Cardiovascular disease (CVD) is a major and growing global social and public healthcare problem, accounting for nearly 50% all deaths in industrialized countries and about 25% in other countries [1]. And CVD has an influence on more than 50% of men and 40% of women over their lifetimes [2]. Measures for prevention of traditional risk factors, including hypertension [3], diabetes mellitus [4], dyslipidemia [4], nondiabetic degrees of hyperglycemia [5] and smoking [6], can be helpful. Investigators have postulated that microalbuminuria may be a marker of risk even in apparently healthy people because it reflects vascular damage in the kidneys and systemic endothelial dysfunction (the Steno hypothesis) [7]. Nowadays, several studies [8, 9] have assumed albuminuria as an independent predictor of major cardiovascular disease (CAD) and end-stage renal disease (ESRD). Albuminuria could be a potential target for intervention to improve major cardiovascular outcome. The urine albumin to creatinine ratio (ACR) is commonly used as an index of albuminuria. An increased urinary albumin excretion is generally defined as ACR ≥ 30 mg/g. Besides, previous study [10] has confirmed that ACR levels of 10 mg/g and 30 mg/g are associated with 1.2 and 1.6 fold relative risks of all-cause mortality compared with ACR level of 5 mg/g. Thus it is of interest to know whether albuminuria below thresholds or above the “overt albuminuria” has indication for cardiovascular events and all-cause mortality.

Elder people are found to be more easily to have kidney damaged evidenced by the presence of elevated levels of albumin. Previous study has demonstrated that elder people has significant higher levels of ACR than the youngest group [11]. We may probably infer that elder people are prone to higher CAD or ESRD risks than the younger individuals. Data on age-related outcomes based on controlled clinical studies is sparse, and little is known about whether the aged people with different levels of ACR have varieties degree of prediction of major cardiovascular outcome.

To investigate the mentioned problems, we undertook a post hoc analysis of a cohort combined with Chinese patients of high cardiovascular risks, to examine 1) the relationship of different level of ACR and cardiovascular outcome; 2) the ACR subgroups stratified by age in influencing cardiovascular events and all-cause mortality.

## Methods

### Study participants

From January 2002 to November 2007, 1474 consecutive hypertensive patients over 55 years with high cardiovascular risks in 33 Chinese hospitals were enrolled in this retrospective study. In detail, patients included in the study had a history of coronary artery disease, peripheral vascular disease, cerebrovascular disease or diabetes mellitus with end organ damage. And in addition, the patients include in our analysis had complete information on age, urinary albumin to creatinine ratio. Exclusion criteria included a serum creatinine level greater than 265 μmol/L (3 mg/dL), clinically significant renal artery stenosis, uncontrolled hypertension (blood pressure > 160 mmHg systolic or > 100 mmHg diastolic), and heart failure. After admission, patients received subsequent medication treatments according to local clinical circumstances back then. Study protocol was approved by Ethics Committee of Fuwai hospital. And all participants provided written informed consent.

### Data collection

Baseline characteristics included basic information, medical history, risk factors, medications of complications and laboratory results. And ACR was calculated as urine albumin to creatinine ratio, and the two variables were measured centrally by an enzymatic immunoassay turbid metric method (Unicel DxC600 Synchron Systems; Beckman Coulter, Brea, California) and a modified Jaffe method (Unicel DxC600 Synchron Systems) respectively. An ACR value between 30 and 299 mg/g was defined as microalbuminuria and a value of 300 mg and over was defined was macro albuminuria. 30 mg/g was the threshold of ACR. Creatinine excretion rate (eGFR) was estimated according to the CKD-EPI formula: a*(serum creatinine/b)^c^ *(0.933)^age^ (a:144 if female, 141 if male), (b:0.7 if female,0.9 if male),(c: for female, if serum creatinine≤0.7 mg/dl, c = − 0.329, if serum creatinine> 0.7 mg/dl, c = − 1.209; for male, if serum creatinine≤0.9 mg/dl, c = − 0.411, if serum> 0.9 mg/dl, c = − 1.209).

### Outcome definitions

In our analyses, the primary endpoint were all-cause mortality and major adverse cardiovascular events (MACE). The secondary endpoints included cardiovascular death, non-fatal myocardial infarction, non-fatal stroke and hospitalization of congestive heart failure. The definitions of different cardiac events were as following: MACE was defined as composite of cardiovascular death, non-fatal myocardial infarction, non-fatal stroke or hospitalization of congestive heart failure. Cardiovascular death was defined as a death for which a definite non-cardiovascular cause (e.g. cancer) has not been identified. Uncertain causes of deaths were presumed to be cardiovascular. Myocardial infarction was followed the universal definition. Stroke was defined as the presence of acute focal neurological deficit thought to be of vascular origin with signs and symptoms lasting greater than 24 h and were confirmed by computed tomography scan or magnetic resonance imaging. Hospitalization for heart failure was defined as overnight hospitalization or attendance in an acute care setting for two of the three following criteria: 1) signs or symptoms of heart failure; 2) radiologic evidence of congestive heart failure; 3) requiring intravenous or a first dose or increased dose of oral diuretic, intravenous or oral vasodilator and intravenous or oral inotrope.

### Statistical analyses

Univariate statistics were reported in means ± standard deviations or counts with proportions as appropriate. Differences in characteristics between different age groups were calculated with Chi-square test for categorical data and student t test for continuous data. Cox proportional hazard models were used to estimate the unadjusted or adjusted hazard rates for death, heart failure or other cardiovascular diseases through strata of ACR, stratified by age. In our analysis, urinary ACR was calculated as a categorical variable (<=10 mg/g, 10.01–30,> 30), with the lowest category (< 10 mg/g) serving as the reference. And further categorization of ACR did not yield different results. Model 1 was adjusted for sex, heart rates, systolic blood pressure, prior myocardial infarction (yes/no), diabetes (yes/no), stroke/transient ischemic attack (TIA) (yes/no), snoring (yes/no), alcohol consumption (yes/no), smoking (yes/no), physical activity (yes/no). Model 2 was adjusted for variables in model 1 plus diastolic blood pressure, statin (yes/no), diuretics (yes/no), angiotensin converting enzyme inhibitor or angiotensin II receptor antagonist (yes/no), eGFR (yes/no). Forward stepwise P for interaction of age and ACR was calculated according to binary logistic analysis. A two-sided *P*-value of 0.05 was considered statistically significant for all analyses, which were conducted using SPSS software version 19.

## Results

### Baseline characteristics

Table 1 shows baseline characteristics of the 1474 participants, the mean age was 65.4 ± 6.5 years and 70.8% were male. The average blood pressure at run-in period was 141/80 mmHg, and resting heart rates was 73 beats per minute. The proportions of myocardial infarction, hypertension and diabetes mellitus were 34.6, 75.0 and 34.3%, respectively. People above 65 and below 65 were determined in 747 and 727 patients. The elder group had more males than the younger group (*p* = 0.036). Elder group had more medication of ASA (*P* = 0.002), clopidogrel (*p* = 0.037) and diuretics (*p* = 0.012) than younger group. And BMI was higher in the younger group compared with the elder one. There was no significant difference in cardiac history, hypertension and diabetes between two groups.

**Table 1:** Baseline Characteristics of All and Age subgroups

Variable	≤65	> 65	*P* value	All
Number of patients, n	747	727		1474
Age, y	60.2 ± 3.4	70.8 ± 3.9	0.000	65.4 ± 6.5
Male, %	68.8%	72.8%	0.036	70.8%
*History, %*				
MI	34.0%	35.2%	0.636	34.6%
Stable angina	19.6%	22.1%	0.095	20.8%
Unstable angina	10.7%	13.2%	0.128	11.9%
CABG	2.0%	2.8%	0.674	2.4%
PTCA/PCI	19.5%	22.7%	0.078	21.1%
Stroke/TIA	53.0%	49.5%	0.336	51.3%
Carotid endarterectomy	0	0		0
Peripheral artery surgery	0.3%	0.7%	0.501	0.5%
Intermittent claudication	2.1%	2.6%	0.915	2.4%
*Risk factors, %*				
Hypertension	74.4%	75.5%	0.944	75.0%
DM	32.8%	35.8%	0.373	34.3%
Current Smoking	15.1%	15.1%	0.741	15.1%
Former smoking	32.0%	33.6%	0.842	32.8%
*Medications, %*				
ACE inhibitors	37.5%	34.7%	0.043	36.1%
Angiotensin II blockers	3.1%	4.4%	0.072	3.7%
β-blockers	37.9%	40.6%	0.271	39.2%
Diuretics	14.2%	19.3%	0.012	16.7%
Nitrates	43.8%	43.9%	0.897	43.8%
Diltiazem/verapamil	9.6%	8.9%	0.933	9.3%
Other calcium-channel blockers	46.3%	45.1%	0.313	45.7%
ASA	65.3%	74.8%	0.002	70.0%
Ticlopidine	2.3%	2.2%	0.766	2.2%
Clopidogrel	2.5%	4.1%	0.037	3.3%
Oral anticoagulants	4.0%	5.8%	0.114	4.9%
statins	26.1%	26.0%	0.812	26.1%
Insulin	6.3%	7.0%	0.829	6.6%
Oral hypoglycemic	24.6%	25.7%	0.617	25.2%
Estrogen(in females)	1.3%	1.5%	0.788	1.4%
Estrogen+progesterone(in females)	0	0.5%	0.282	0.2%
*Physical exam*				
Heart rate (beats/min)	73	73	0.085	73
BP at run-in(mmHg)	140/80	141/80	0.261/0.761	141/80
BP at randomization(mmHg)	133/77	133/76	0.948/0.341	133/76
BMI	25.9	24.7	0.016	25.4
Waist-hip ratio	0.9	1.0	0.444	0.9
*Laboratory results*				
Creatinine(μmol/L)	87.7	93.5	0.000	90.5
Potassium(mmol/L)	4.4	4.3	0.226	4.4
Total cholesterol(mmol/L)	5.3	5.1	0.044	5.2
HDL-cholesterol(mmol/L)	1.3	1.3	0.989	1.3
LDL-cholesterol(mmol/L)	2.8	2.8	0.949	2.8
Triglycerides(mmol/L)	1.8	1.7	0.067	1.8

### Rates of cardiovascular events and all-cause mortality according to ACR subgroups

**Fig. 1:**
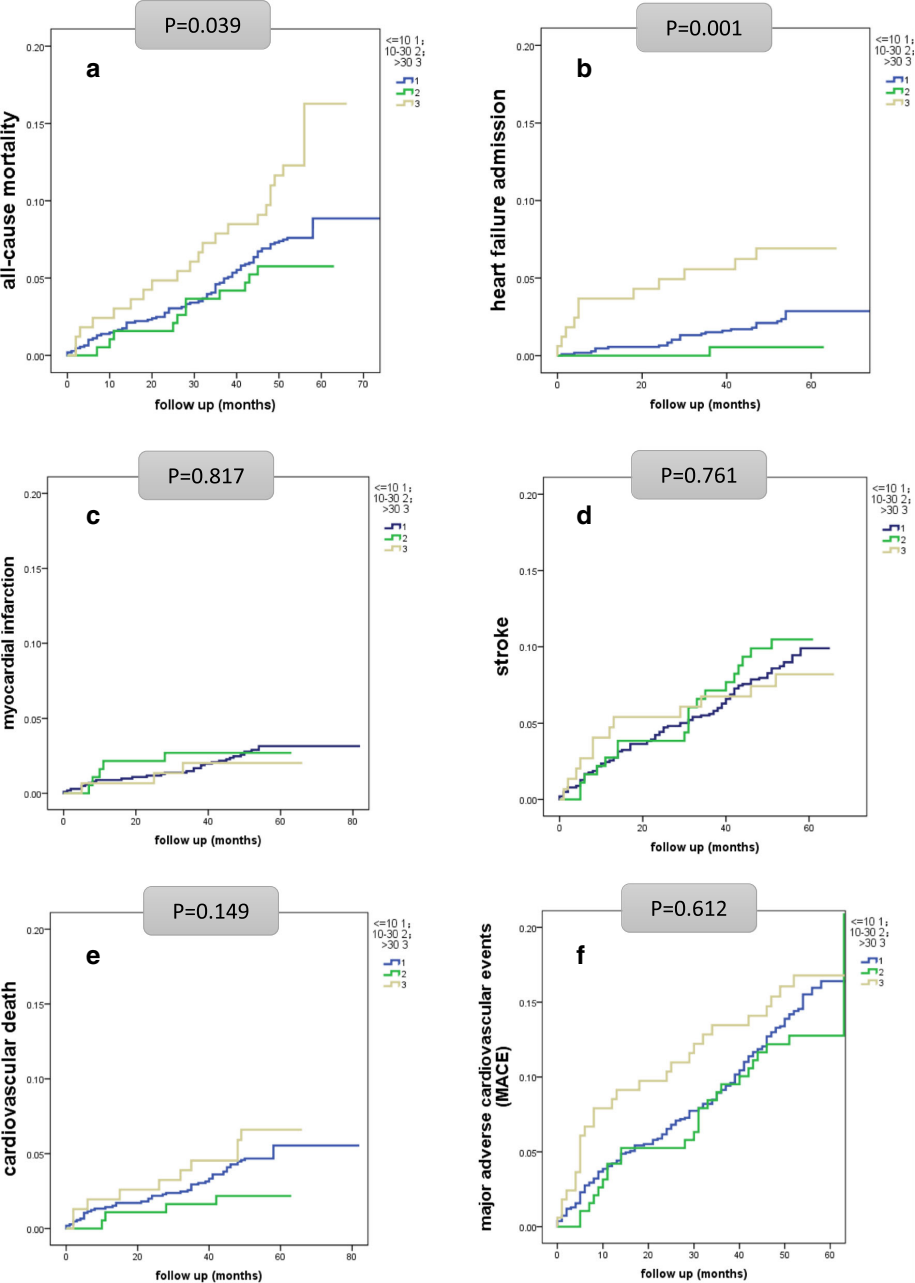
Kaplan-Meier survival event curves for all-cause mortality (**a**), hospitalization of congestive heart failure (**b**), myocardial infarction (**c**), stroke (**d**), cardiovascular death (**e**) and the composite of cardiovascular outcome (**f**), according to three levels of ACR

In Fig. 1, Kaplan-Meier curves summarize the development of all-cause mortality (Fig. 1a), hospitalization of congestive heart failure (Fig. 1b), myocardial infarction (Fig. 1c), stroke (Fig. 1d), cardiovascular death (Fig. 1e), and MACE (Fig. 1f). The outcome of ACR subgroups differ in all-cause mortality and hospitalization of congestive heart failure. And the level of ACR above 30 mg/g had more mortality rates and heart failure admission than the other two groups. However, myocardial infarction, stroke, cardiovascular death and MACE were not statistically significant between different ACR groups.

### The association of ACR and age groups with all-cause mortality

A total of 117 deaths were observed (Table 2). Among all the participants, higher level of ACR was associated with higher mortality rates in unadjusted model, and the hazard ratios (HR) of ACR above 30 mg/g was 1.69 comparing with ACR below 10 mg/g (95% CI 1.05–2.72, *p* = 0.043). While in multivariable-adjusted models, the ACR levels had no connection with all-cause mortality. Then the same analyses were performed in different age groups. And we found that in elder group the ACR levels were associated with mortality rates. In unadjusted models, the risk of death in ACR above 30 mg/g was 2-fold of ACR below 10 mg/g (HR 2.00, 95%CI 1.18–3.41, *p* = 0.008). While in multivariable adjusted models, the HR is 1.95 in higher level of ACR compared with the reference (95% CI 0.99–3.86, *p* = 0.048). To test whether there was an interaction between age and ACR, the respective interaction term was added to all models, and the *P*-values were not statistically significant (*p* > 0.05) for all-cause mortality.

**Table 2:** Hazard ratios associated with ACR and age subgroup for all-cause mortality

Outcome and age subgroups	ACR category				
	≦10 mg/g	10 to 30 mg/g	> 30 mg/g	Total, n or P value^e^	P for interaction^f^
All-cause mortality					
≦65 years					
Events, n	30	5	3	38	
Patients, n	579	92	76	747	
Hazard ratio (95% CI)					
Unadjusted	1(ref)	1.05 (0.41–2.70)	0.76 (0.23–2.50)	0.896	
Adjusted by model 1^a^	1(ref)	0.49 (0.11–2.13)	0.96 (0.27–3.38)	0.637	
Adjusted by model 2^b^	1(ref)	0.48 (0.11–2.10)	0.95 (0.27–3.42)	0.624	
> 65 years					
Events, n	55	6	18	79	
Patients, n	531	104	92	727	
Hazard ratio (95% CI)					
Unadjusted	1(ref)	0.55 (0.24–1.29)	2.00 (1.18–3.41)	0.008	
Adjusted by model 1^a^	1(ref)	0.55 (0.19–1.57)	1.86 (0.94–3.67)	0.061	
Adjusted by model 2^b^	1(ref)	0.56 (0.19–1.61)	1.95 (0.99–3.86)	0.048	
Total					
Events, n	85	11	21	117	
Patients, n	1110	196	168	1474	
Hazard ratio (95% CI)					
Unadjusted	1(ref)	0.73 (0.39–1.38)	1.69 (1.05–2.72)	0.043	0.180^f^
Adjusted by model 3^c^	1(ref)	0.50 (0.21–1.20)	1.45 (0.81–2.60)	0.088	0.594^f^
Adjusted by model 4^d^	1(ref)	0.51 (0.21–1.20)	1.47 (0.82–2.65)	0.082	0.524^f^

ACR indicates albuminuria-to-creatinine ratio

^a^model 1 was adjusted for sex, heart rates, systolic blood pressure, diabetes, prior myocardial infarction, stroke/transient ischemic attack, snoring, alcohol consumption, smoking, physical activity

^b^model 2 was adjusted for sex, heart rates, systolic blood pressure, diastolic blood pressure, diabetes, prior myocardial infarction, stroke/transient ischemic attack, eGFR, statin, diuretics, angiotensin converting enzyme inhibitor or angiotensin II receptor antagonist, snoring, alcohol consumption, smoking, physical activity

^c^model 3 was adjusted model 1 plus age. ^d^model 4 was adjusted model 2 plus age

^e^P indicates the comparison between different ACR categories

^f^P for interaction indicates the interaction between ACR categories and age subgroups

### The association of ACR and age groups with cardiovascular events

The results of cardiovascular death, non-fatal myocardial infarction, non-fatal stroke and hospitalization of congestive heart failure, and the composite event---MACE were presented in Table 3 and Additional file 1: Table S1-S4. Only hospitalization of congestive heart failure was demonstrated a statistically significant with regard to different levels of ACR, especially in high and low levels of ACR, since patients who had the medium level of ACR (10 to 30 mg/g) were scarce. In unadjusted models, the HR of higher levels of ACR (> 30 mg/g) was nearly 3-fold of the reference ACR (HR 2.95, 95%CI 1.46–5.96, *p* = 0.003). After being adjusted, the strength of the association was exaggerated (HR 4.58, 95%CI 1.78–11.76, *p* = 0.007). In patients below the age of 65, compared with lower level of ACR (< 10 mg/g), ACR levels of > 30 mg/g were associated with around 11-fold independent risk of heart failure hospitalization (HR 11.22, *p* = 0.033). While the risk reduced to nearly 6-fold in patients above the age of 65 (HR 5.85, *p* = 0.002).

**Table 3:** Hazard ratios associated with ACR and age subgroup for hospitalization of congestive heart failure

Outcome and age subgroups	≦10 mg/g	10 to 30 mg/g	> 30 mg/g	Total, n or P value^e^	P for interaction^f^
Heart failure admission					
≦65 years					
Events, n	5	0	5	10	
Patients, n	579	92	76	747	
Hazard ratio (95% CI)					
Unadjusted	1(ref)	–	7.76 (2.25–26.79)	0.001	
Adjusted by model 1^a^	1(ref)	–	15.37 (1.82–130.03)	0.012	
Adjusted by model 2^b^	1(ref)	–	11.22 (1.21–104.30)	0.033	
> 65 years					
Events, n	21	1	7	29	
Patients, n	531	104	92	727	
Hazard ratio (95% CI)					
Unadjusted	1(ref)	0.24 (0.03–1.77)	1.76 (0.71–4.37)	0.146	
Adjusted by model 1^a^	1(ref)	–	5.70 (1.84–17.63)	0.003	
Adjusted by model 2^b^	1(ref)	–	5.85 (1.87–18.35)	0.002	
Total					
Events, n	26	1	12	39	
Patients, n	1110	196	168	1474	
Hazard ratio (95% CI)					
Unadjusted	1(ref)	0.22(0.03–1.60)	2.95 (1.46–5.96)	0.003	0.529^f^
Adjusted by model 3^c^	1(ref)	–	5.21 (2.05–13.22)	0.001	0.668^f^
Adjusted by model 4^d^	1(ref)	–	4.58 (1.78–11.76)	0.007	0.814^f^

ACR indicates albuminuria-to-creatinine ratio

^a^model 1 was adjusted for sex, heart rates, systolic blood pressure, diabetes, prior myocardial infarction, stroke/transient ischemic attack, snoring, alcohol consumption, smoking, physical activity

^b^model 2 was adjusted for sex, heart rates, systolic blood pressure, diastolic blood pressure, diabetes, prior myocardial infarction, stroke/transient ischemic attack, eGFR, statin, diuretics, angiotensin converting enzyme inhibitor or angiotensin II receptor antagonist, snoring, alcohol consumption, smoking, physical activity

^c^model 3 was adjusted model 1 plus age. ^d^ model 4 was adjusted model 2 plus age

^e^P indicates the comparison between different ACR categories

^f^P for interaction indicates the interaction between ACR categories and age subgroups

In multivariable adjusted models, different levels of ACR were not statistically significant in MACE, cardiovascular death, myocardial infarction and stroke. Similarly, the conclusion did not change when stratified by age groups.

## Discussion

Our study found that the value of ACR was associated with higher all-cause mortality and admission for heart failure in Chinese patients with high cardiovascular risks. Increased ACR was found to be independently associated with all-cause mortality in univariate analysis, but the association disappeared after adjusting for multivariable factors. However, we found that elder patients (above the age of 65) with increased ACR tended to experience higher mortality risks, even after adjustment in multivariable models. Moreover, in our analysis, increased ACR was associated with higher hospitalization of congestive heart failure in either younger or elder age groups. And the association seemed to be more prominent among patients below the age of 65 than those above 65.

However, the primary endpoint--MACE, cardiovascular death, stroke and myocardial infarction showed no difference in three categories of urinary ACR (> 30 mg/g, 10 mg/g < ACR ≤ 30 mg/g, ≤10 mg/g) in our cohort, which differs from the previously published analyses of several trials [12, 13]. The probable reason could be limit of sample size, and the characteristic features of this Chinese cohort. In other trials, reduction in albuminuria relating to the reduction in cardiovascular events had been demonstrated in certain individuals such as patients with diabetic nephropathy, hypertension with left ventricular hypertrophy, patients without hypertension or diabetic mellitus as well as patients with prior MI or stroke [14]. The lack of benefit on the cardiovascular death, stroke and myocardial infarction in the patients with normal albuminuria was somewhat surprising. Although our findings were unexpected, the data were robust. In our analyses, ACR could be an independent risk factor of all-cause mortality. In meta-analyses conducted by the Chronic Kidney Disease Prognosis Consortium (CKD-PC) [10] had established the elaborate assessment of the independent and combined association of eGFR and albuminuria with mortality. And the study showed that the association between albuminuria and mortality was linear. In our analysis, we chose group-dividing method similar as CKD-PC, and used reference group as 10 mg/g instead of 5 mg/g mainly due to limited sample size of our study. And we found a 1.69-fold higher risk of all-cause mortality in high grade of albuminuria (> 30 mg/g) compared with the reference albuminuria (≤10 mg/g).

In secondary outcome, ACR was also independently associated with heart failure admission. In our study, participants with high grade of baseline albuminuria (> 30 mg/g) had a 2.95-fold higher risk of heart failure admission compared with patients with low grade albuminuria (≤10 mg/g). In the adjusted risk models, the HR was exaggerated to 4 to 5 fold. Similar results were found in several studies, in which the influence of ACR to heart failure admission was not as strong as our study. The reason was probably that our study included patients with more cardiovascular risks, such as hypertension and diabetes mellitus. And this population was more inclined to have higher level of baseline albuminuria. However, no analyses of age subgroups were mentioned in these studies. Our subgroup analyses divided participants into groups split at the age of 65, and HRs tended to be higher in patients below the age of 65 (hazard ratio 7.67,95%CI 2.13–27.64, *P* = 0.008; comparing ACR > 30 mg/g with ACR ≤ 10 mg/g) than above 65. The reason of this phenomenon may be due to higher mortality rates among elderly, which could attenuate the effect of heart failure admission, thus increased ACR alone seemed less significant.

We also found that age was an effect factor for ACR, and several studies had shown the effect of all-cause mortality was modified by age. In our analyses, ACR levels were more associated with all-cause mortality in “elder” participants. O’Hare and colleagues reported ACR levels ≥30 mg/g to be associated with increased mortality among adults≥75 years of age [15]. Additionally, in general population and high risk cohort included in the Chronic Kidney Disease Prognosis Consortium (CKD-PC), the hazard ratio for all-cause mortality associated with higher ACR levels (42.5 mg/g versus. 5.1 mg/g) was similar for adults< 65 years of age (HR 1.49, 95%CI 1.40–1.59) and ≥ 65 years of age (HR 1.52,95%CI 1.45–1.61) [10]. Compared with the study of CKD-PC, this Chinese cohort enrolled participants with high cardiovascular risks and in other comparison of the age group, hazard ratios for mortality were present no statistically significant. In spite of this, the absolute differences in mortality rated stratified by level of ACR and age were higher at microalbuminuria and elder patients. And interaction between age and ACR with mortality was not present.

Our study did not found the connection between medium grade of albuminuria (10 mg/g < ACR ≤ 30 mg/g) had prediction effect of cardiovascular events. Previous research yielded inconsistent results with regards to the prognostic significance of levels of albuminuria below the microalbuminuria threshold. In European Prospective Investigation into Cancer in Norfolk (EPIC-Norfolk) study, low grade albuminuria was not associated with an increased risk of coronary heart disease, but microalbuminuria was [16]. In the Nord-Trondelag Health (HUNT) study, low grade albuminuria (> 6.7μg/mg) was associated with increased all-cause mortality, however, that study ignored to evaluate the incidence of CVD. Moreover, the HUNT study was conducted with a mailed questionnaire and an invitation to clinical examination, thus the study sample included untreated hypertensive patients. And exclusion of hypertensive or diabetic individuals attenuated the risk associated with low grade albuminuria [17]. On the contrast, in the Prevention of Renal and Vascular End stage Disease (PREVEND) study, a more graded increase in vascular mortality was observed through the entire range of urinary ACR [14]. Similar results were found in The Heart Outcomes Prevention Evaluation (HOPE) study, in which the risk starting well below the microalbuminuria cutoff. In a cohort of individuals with non-hypertension or non-diabetics showed low grade urinary albumin excretion was associated with increased risk of CVD and mortality, though the increase was modest in terms of the absolute event rates [7]. According to the finding of our study, ACR ≥ 30 mg/g could be an indication of increased mortality risk, which was consistent with the current KDOQI threshold. Though an optimum ACR level was associated with a lower mortality rate and heart failure admission, definition of threshold of ACR should incorporate various considerations, such as the prevalence of the disease, the specific outcomes, and even cost-effectiveness of public health.

The connection of ACR and poor cardiovascular outcome can be explained in various aspects. First, albuminuria has been verified as a well-recognized marker of systemic microvascular injury and has been associated with subclinical myocardial dysfunction in individuals with normal LVEF. It is likely that albuminuria represents a sensitive marker of early dysregulation in cardio renal axis and thus albuminuria associates with early declines in cardiac function and increases cardiovascular disease. Second, albuminuria is associated with an arterial dysfunction not limited to the glomerulus and other micro vessels but also present in conduit arteries. Measurement of albuminuria may therefore contribute to estimation of vascular status and cardiovascular risk. Third, Albuminuria may occur as a result of direct transmission of raised systemic pressure to the glomerulus and perm selectivity changes of the glomerular filter. Finally, inflammation is implicated in early endothelial dysfunction and more advanced atherosclerosis, which indicated impaired autoregulation of glomerular pressure and dysfunction of the glomerular endothelium. These factors may enhance albuminuria [18–21].

The results of this study should be interpreted within the context of certain limitations. Patients enrolled in this analyses were Chinese elder patients with high cardiovascular risks, thus limiting the generalizability of our results to other ethnicities and younger individuals. Patients enrolled in these clinical trials were thoughtfully selected that those with extremely high albuminuria and severe renal dysfunction were excluded. In addition, urinary albumin was assessed on a single urine specimen in this study, and any potential measurement error would result in an underestimation of the true risk associated with urinary ACR. Furthermore, urinary albumin levels may have considerable intra-individual variability. Moreover, the number of events occurring in our sample was small, as would be explained by its low risk nature. Consequently, we had too few events in those with urinary ACR values below the threshold of microalbuminuria to examine this group separately.

## Conclusion

Our observations support that higher urinary ACR is associated with higher all-cause mortality and admission of heart failure in Chinese individuals with high cardiovascular risks. Patients above the age of 65 with increased ACR tended to experience higher mortality risks. This observation is a post hoc comparison and should be interpreted appropriately. Further studies are needed to find out whether there is age-specific ACR cutoff point, and fully appreciation of the relationship between ACR and cardiovascular events and mortality across a broad age range.

## Additional file

Additional file 1: **Table S1.** Hazard ratios associated with ACR and age subgroup for major adverse cardiovascular event (MACE). **Table S2.** Hazard ratios associated with ACR and age subgroup for cardiovascular death. **Table S3.** Hazard ratios associated with ACR and age subgroup for stroke. **Table S4.** Hazard ratios associated with ACR and age subgroup for myocardial infarction. (DOCX 38 kb)
