# Supplementary material for: Albumin-to-creatinine ratio as a predictor of all-cause mortality and hospitalization of congestive heart failure in Chinese elder hypertensive patients with high cardiovascular risks
Source: Clin Hypertens. 2018 Aug 15;24:12. doi: 10.1186/s40885-018-0095-3 (PMC6109984; doi:10.1186/s40885-018-0095-3)
Supplement: Supplementary file 1 — Table S1. Hazard ratios associated with ACR and age subgroup for major adverse cardiovascular event (MACE). Table S2. Hazard ratios associated with ACR and age subgroup for cardiovascular death. Table S3. Hazard ratios associated with ACR and age subgroup for stroke. Table S4. Hazard ratios associated with ACR and age subgroup for myocardial infarction. (DOCX 38 kb) [file 40885_2018_95_MOESM1_ESM.docx]

Table S1 Hazard ratios associated with ACR and age subgroup for major adverse cardiovascular event (MACE).

| Outcome and age subgroups | ACR categroy | | | | |
| --- | --- | --- | --- | --- | --- |
|  | **≦10mg/g** | **10 to ≦30 mg/g** | **>30mg/g** | **Total, n or P value ‡** | P for interaction § |
| MACE |  |  |  |  |  |
| ≦65 years |  |  |  |  |  |
| Events, n | 68 | 12 | 9 | 89 |  |
| Patients, n | 579 | 92 | 76 | 747 |  |
| Hazard ratio(95% CI) |  |  |  |  |  |
| Unadjusted | 1(ref) | 1.13 (0.61-2.08) | 1.03 (0.51-2.05) | 0.930 |  |
| Adjusted by model 1* | 1(ref) | 1.32 (0.64-2.76) | 0.92 (0.38-2.24) | 0.719 |  |
| Adjusted by model 2† | 1(ref) | 1.32 (0.63-2.76) | 0.85 (0.34-2.09) | 0.673 |  |
| >65 years |  |  |  |  |  |
| Events, n | 93 | 13 | 18 | 124 |  |
| Patients, n | 531 | 104 | 92 | 727 |  |
| Hazard ratio(95% CI) |  |  |  |  |  |
| Unadjusted | 1(ref) | 0.70 (0.39-1.26) | 1.20 (0.73-1.99) | 0.333 |  |
| Adjusted by model 1* | 1(ref) | 0.38 (0.14-1.07) | 1.60 (0.85-3.03) | 0.040 |  |
| Adjusted by model 2† | 1(ref) | 0.39 (0.14-1.08) | 1.64 (0.87-3.11) | 0.038 |  |
| Total |  |  |  |  |  |
| Events, n | 161 | 25 | 27 | 213 |  |
| Patients, n | 1110 | 196 | 168 | 1474 |  |
| Hazard ratio(95% CI) |  |  |  |  |  |
| Unadjusted | 1(ref) | 0.88 (0.58-1.35) | 1.16 (0.77-1.74) | 0.614 | 0.495 § |
| Adjusted by model 3** | 1(ref) | 0.70 (0.39-1.25) | 1.20 (0.72-1.99) | 0.315 | 0.292 § |
| Adjusted by model 4†† | 1(ref) | 0.70 (0.38-1.25) | 1.20 (0.72-1.99) | 0.314 | 0.293 § |

ACR indicates albuminuria-to-creatinine ratio

* model 1 was adjusted for sex, heart rates, systolic blood pressure, diabetes, prior myocardial infarction, stroke/transient ischemic attack, snoring, alcohol consumption, smoking, physical activity

† model 2 was adjusted for sex, heart rates, systolic blood pressure, diastolic blood pressure, diabetes, prior myocardial infarction, stroke/transient ischemic attack, eGFR, statin, diuretics, angiotensin converting enzyme inhibitor or angiotensin II receptor antagonist, snoring, alcohol consumption, smoking, physical activity.

** model 3 was adjusted model 1 plus age. †† model 4 was adjusted model 2 plus age.

‡ P indicates the comparison between different ACR categories.

§ P for interaction indicates the interaction between ACR categories and age subgroups.

Table S2 Hazard ratios associated with ACR and age subgroup for cardiovascular death.

| Outcome and age subgroups | ACR category | | | | |
| --- | --- | --- | --- | --- | --- |
|  | **≦10mg/g** | **10 to ≦30 mg/g** | **>30mg/g** | **Total, n or P value ‡** | P for interaction § |
| Cardiovascular death |  |  |  |  |  |
| ≦65 years |  |  |  |  |  |
| Events, n | 22 | 2 | 2 | 26 |  |
| Patients, n | 579 | 92 | 76 | 747 |  |
| Hazard ratio(95% CI) |  |  |  |  |  |
| Unadjusted | 1(ref) | 1.44 (0.34-6.14) | 0.83 (0.12-5.91) | 0.688 |  |
| Adjusted by model 1* | 1(ref) | --- | 0.83 (0.18-3.85) | 0.973 |  |
| Adjusted by model 2† | 1(ref) | --- | 0.86 (0.17-4.20) | 0.983 |  |
| >65 years |  |  |  |  |  |
| Events, n | 29 | 2 | 8 | 39 |  |
| Patients, n | 531 | 104 | 92 | 727 |  |
| Hazard ratio(95% CI) |  |  |  |  |  |
| Unadjusted | 1(ref) | 0.35 (0.08-1.45) | 1.76 (0.80-3.85) | 0.098 |  |
| Adjusted by model 1* | 1(ref) | 0.29 (0.04-2.25) | 2.00 (0.71-5.61) | 0.153 |  |
| Adjusted by model 2† | 1(ref) | 0.31 (0.04-2.38) | 2.35 (0.84-6.58) | 0.100 |  |
| Total |  |  |  |  |  |
| Events, n | 51 | 4 | 10 | 65 |  |
| Patients, n | 1110 | 196 | 168 | 1474 |  |
| Hazard ratio(95% CI) |  |  |  |  |  |
| Unadjusted | 1(ref) | 0.45 (0.16-1.23) | 1.37 (0.69-2.69) | 0.166 | 0.336 § |
| Adjusted by model 3** | 1(ref) | 0.16 (0.02-1.15) | 1.35 (0.59-3.08) | 0.128 | 0.278 § |
| Adjusted by model 4†† | 1(ref) | 0.16 (0.02-1.18) | 1.48 (0.64-3.41) | 0.109 | 0.297 § |

ACR indicates albuminuria-to-creatinine ratio

* model 1 was adjusted for sex, heart rates, systolic blood pressure, diabetes, prior myocardial infarction, stroke/transient ischemic attack, snoring, alcohol consumption, smoking, physical activity

† model 2 was adjusted for sex, heart rates, systolic blood pressure, diastolic blood pressure, diabetes, prior myocardial infarction, stroke/transient ischemic attack, eGFR, statin, diuretics, angiotensin converting enzyme inhibitor or angiotensin II receptor antagonist, snoring, alcohol consumption, smoking, physical activity.

** model 3 was adjusted model 1 plus age. †† model 4 was adjusted model 2 plus age.

‡ P indicates the comparison between different ACR categories.

§ P for interaction indicates the interaction between ACR categories and age subgroups.

Table S3 Hazard ratios associated with ACR and age subgroup for stroke.

| Outcome and age subgroups | ACR category | | | | |
| --- | --- | --- | --- | --- | --- |
|  | **≦10mg/g** | **10 to ≦30 mg/g** | **>30mg/g** | **Total, n or P value ‡** | P for interaction § |
| stroke |  |  |  |  |  |
| ≦65 years |  |  |  |  |  |
| Events, n | 39 | 11 | 4 | 54 |  |
| Patients, n | 579 | 92 | 76 | 747 |  |
| Hazard ratio(95% CI) |  |  |  |  |  |
| Unadjusted | 1(ref) | 1.85 (0.95-3.62) | 0.79 (0.28-2.22) | 0.154 |  |
| Adjusted by model 1* | 1(ref) | 2.17 (0.98-4.80) | 0.69 (0.20-2.43) | 0.102 |  |
| Adjusted by model 2† | 1(ref) | 2.44 (1.09-5.50) | 0.65 (0.18-2.31) | 0.052 |  |
| >65 years |  |  |  |  |  |
| Events, n | 53 | 8 | 8 | 69 |  |
| Patients, n | 531 | 104 | 92 | 727 |  |
| Hazard ratio(95% CI) |  |  |  |  |  |
| Unadjusted | 1(ref) | 0.75 (0.36-1.57) | 0.98 (0.47-2.06) | 0.744 |  |
| Adjusted by model 1* | 1(ref) | 0.31 (0.07-1.28) | 1.00 (0.37-2.68) | 0.264 |  |
| Adjusted by model 2† | 1(ref) | 0.31 (0.07-1.30) | 0.98 (0.36-2.66) | 0.272 |  |
| Total |  |  |  |  |  |
| Events, n | 92 | 19 | 12 | 123 |  |
| Patients, n | 1110 | 196 | 168 | 1474 |  |
| Hazard ratio(95% CI) |  |  |  |  |  |
| Unadjusted | 1(ref) | 1.18 (0.72-1.93) | 0.92 (0.50-1.68) | 0.762 | 0.167 § |
| Adjusted by model 3** | 1(ref) | 1.01 (0.53-1.94) | 0.83 (0.38-1.82) | 0.895 | 0.089 § |
| Adjusted by model 4†† | 1(ref) | 1.03 (0.53-1.97) | 0.83 (0.38-1.81) | 0.884 | 0.088 § |

ACR indicates albuminuria-to-creatinine ratio

* model 1 was adjusted for sex, heart rates, systolic blood pressure, diabetes, prior myocardial infarction, stroke/transient ischemic attack, snoring, alcohol consumption, smoking, physical activity

† model 2 was adjusted for sex, heart rates, systolic blood pressure, diastolic blood pressure, diabetes, prior myocardial infarction, stroke/transient ischemic attack, eGFR, statin, diuretics, angiotensin converting enzyme inhibitor or angiotensin II receptor antagonist, snoring, alcohol consumption, smoking, physical activity.

** model 3 was adjusted model 1 plus age. †† model 4 was adjusted model 2 plus age.

‡ P indicates the comparison between different ACR categories.

§ P for interaction indicates the interaction between ACR categories and age subgroups.

Table S4 Hazard ratios associated with ACR and age subgroup for myocardial infarction.

| Outcome and age subgroups | ACR category | | | | |
| --- | --- | --- | --- | --- | --- |
|  | **≦10mg/g** | **10 to ≦30 mg/g** | **>30mg/g** | **Total, n or P value ‡** | P for interaction § |
| Myocardial infarction |  |  |  |  |  |
| ≦65 years |  |  |  |  |  |
| Events, n | 14 | 1 | 0 | 15 |  |
| Patients, n | 579 | 92 | 76 | 747 |  |
| Hazard ratio(95% CI) |  |  |  |  |  |
| Unadjusted | 1(ref) | 0.44 (0.06-3.38) | --- | 0.735 |  |
| Adjusted by model 1* | 1(ref) | --- | --- | --- |  |
| Adjusted by model 2† | 1(ref) | --- | --- | --- |  |
| >65 years |  |  |  |  |  |
| Events, n | 16 | 4 | 3 | 23 |  |
| Patients, n | 531 | 104 | 92 | 727 |  |
| Hazard ratio(95% CI) |  |  |  |  |  |
| Unadjusted | 1(ref) | 1.22 (0.41-3.64) | 1.18 (0.35-4.06) | 0.920 |  |
| Adjusted by model 1* | 1(ref) | 1.06 (0.21-5.34) | 2.20 (0.50-9.61) | 0.563 |  |
| Adjusted by model 2† | 1(ref) | 1.08 (0.22-5.45) | 2.20 (0.49-9.94) | 0.580 |  |
| Total |  |  |  |  |  |
| Events, n | 30 | 5 | 3 | 38 |  |
| Patients, n | 1110 | 196 | 168 | 1474 |  |
| Hazard ratio(95% CI) |  |  |  |  |  |
| Unadjusted | 1(ref) | 0.92 (0.37-2.36) | 0.69 (0.21-2.25) | 0.819 | 0.043 § |
| Adjusted by model 3** | 1(ref) | 0.67 (0.15-3.00) | 1.21 (0.33-4.48) | 0.811 | 0.049 § |
| Adjusted by model 4†† | 1(ref) | 0.65 (0.15-2.93) | 1.12 (0.30-4.20) | 0.825 | 0.055 § |

ACR indicates albuminuria-to-creatinine ratio

* model 1 was adjusted for sex, heart rates, systolic blood pressure, diabetes, prior myocardial infarction, stroke/transient ischemic attack, snoring, alcohol consumption, smoking, physical activity

† model 2 was adjusted for sex, heart rates, systolic blood pressure, diastolic blood pressure, diabetes, prior myocardial infarction, stroke/transient ischemic attack, eGFR, statin, diuretics, angiotensin converting enzyme inhibitor or angiotensin II receptor antagonist, snoring, alcohol consumption, smoking, physical activity.

** model 3 was adjusted model 1 plus age. †† model 4 was adjusted model 2 plus age.

‡ P indicates the comparison between different ACR categories.

§ P for interaction indicates the interaction between ACR categories and age subgroups.
